# High state boredom vastly affects psychiatric inpatients and predicts their treatment duration

**DOI:** 10.1038/s41398-023-02650-9

**Published:** 2023-11-16

**Authors:** Johannes P.-H. Seiler, Katharina Zerr, Simon Rumpel, Oliver Tüscher

**Affiliations:** 1 Institute of Physiology, Focus Program Translational Neurosciences, University Medical Center of the Johannes Gutenberg University Mainz, Hanns-Dieter-Hüsch-Weg 19, 55131 Mainz, Germany; 2 Department of Psychiatry and Psychotherapy, University Medical Center of the Johannes Gutenberg University Mainz, Untere Zahlbacher Straße 8, 55131 Mainz, Germany; 3 Leibniz Institute for Resilience Research, Wallstraße 7, 55122 Mainz, Germany; 4 Institute of Molecular Biology, Ackermannweg 4, 55128 Mainz, Germany

**Keywords:** Neuroscience, Psychiatric disorders

## Abstract

Boredom is a ubiquitous, aversive human experience typically elicited by low information and monotony. Boredom can occur either as a transient mental state that prompts individuals to adapt their behavior to avoid monotony or as a temporally stable trait, describing a chronic susceptibility to feeling bored. Increased trait boredom was found to correlate with various psychopathologies and indicators of mental burden. However, the role of state boredom in psychopathological conditions and its implications for psychiatric treatment remain elusive. Here, we address this issue by investigating state boredom and trait boredom in a cohort of psychiatric inpatients and a healthy control cohort. We find that in both groups, state boredom, even more than trait boredom, shows remarkable associations with psychopathology. In the inpatient group, state boredom is implicated broadly in multiple mental disorders and shows an association with treatment in closed psychiatric wards. Furthermore, through statistical modeling, we find that high-state boredom during inpatient therapy is predictive of a longer therapy duration. Thus, we show that state boredom constitutes an indicator of mild and severe psychopathology in different mental disorders, affecting the outcome of psychiatric patients. Potential therapeutic interventions are discussed, aiming to enhance information flow in the brain in order to alleviate boredom in clinical settings.

## Introduction

Arising in various situations of everyday life, boredom is an unpleasant human experience that can drive individuals to escape current sources of monotony and increase information flow to the brain [1–4]. As such, boredom has been defined either as a transient mental *state*, elicited by circumscribed environmental or psychological conditions or as a *trait*, describing the general tendency to become bored in a variety of environments [5–8].

Although, state boredom has a more immediate and physiological effect on behavior, e.g., by promoting information-seeking in monotonous environments [1, 4, 5, 9], past research has largely focused on the implications of trait boredom, observing a link to multiple symptoms of psychopathology [10]. More specifically, trait boredom was found to correlate reliably with impulsivity, risk affinity, and sensation-seeking [11] in the context of mental disorders like attention deficit hyperactivity disorder and borderline personality disorder [12–15]. In addition, addictive behaviors like pathological gambling [16–18], alcohol abuse [19, 20] as well as depression [21–24], and psychotic disorders [8, 25, 26] have been linked with increased boredom susceptibility. Even aggressive patient behavior in clinical settings was found to be predicted by boredom [27–29].

Despite these severe psychosocial implications, boredom is still widely neglected in the routines of mental health care [8, 26, 27, 30]. In particular, the role of state boredom in evident psychopathologies and their clinical consequences remains unclear. Thus, a deeper understanding of state boredom in psychiatric disorders seems promising, as it could enable the development of targeted therapeutic interventions to alleviate boredom and improve clinical outcomes [8, 27, 30].

Here, we address this knowledge gap by characterizing state boredom and trait boredom in a broad cohort of psychiatric inpatients and comparing it to the level of boredom measured in a healthy control cohort. First, we replicate correlations of boredom with mild psychopathology in healthy humans and then characterize inpatient boredom for different groups of mental disorders. We assess state boredom over the course of inpatient therapy and find that it generally reduces over time, while this reduction is modulated by the diagnosis group and treatment conditions in open versus closed psychiatric wards. We use boredom together with other clinical variables to predict the duration of therapy for single inpatients and find that state boredom is associated strongly with therapy outcomes.

Thus, we identify state boredom as an important determinant of mild and severe psychopathology in various mental disorders with a notable effect on the success of psychiatric inpatient treatment. We integrate our findings into a framework that defines boredom as a signal of an individual’s need for information and discuss how boredom-related information deficits could be targeted in clinical settings.

## Methods

The study was approved by the local ethics committee (Ethikkommission der Landesärztekammer Rheinland-Pfalz, processing numbers: 2018–13164 and 2018-13164_1) and was conducted in accordance with the Declaration of Helsinki. Written informed consent was obtained from all participants of the study.

### Boredom assessment in a healthy control cohort

To assess exploratively the link between boredom and psychopathological symptoms in a healthy sample, we randomly contacted approximately 1800 subjects from the pool of the *Gutenberg Brain Study* (https://lir-mainz.de/en/gutenberg-brain-study). This study comprises a pool of several thousand adults aged 18–60 who gave consent to be invited to take part in health-related surveys. Subjects were informed about the study by post and asked to participate. From the individuals contacted, we received written consent from 883 individuals we deemed valid for participation in the study. Exclusion criteria were active psychiatric or neurological disorders.

All subjects filled out psychometric questionnaires to assess state boredom (*MSBS*: Multidimensional State Boredom Scale [31], referring to their feeling in the moment of filling out the questionnaire), trait boredom (*BPS*: Boredom Proneness Scale [32]) and characteristics of their mental health status (*GHQ-28*: General Health Questionnaire [33], *CAARS:S-L*: Conner’s Adult ADHD Rating Scale [34], *BDI-II*: Beck’s Depression Inventory [35], *I-8*: Impulsivity Questionnaire [36, 37], *STAI-Y*: State Trait Anxiety Inventory [38]) as well as general information about their sociodemographic background and patient history. Subjects received an expense allowance of 5€ for participation. The demographic characteristics of the healthy cohort are detailed in Supplementary Table 1. The dataset obtained through this survey was further used in a study to validate the German translations of the MSBS and BPS (currently in preparation for publication).

### Boredom assessment in a broad cohort of psychiatric patients

To investigate the implications of state boredom and trait boredom in a clinical sample of psychiatric inpatients, we recruited a broad cohort of people who underwent treatment in a general psychiatric care hospital. For this purpose, all patients who were admitted to the inpatient facilities of the Department of Psychiatry and Psychotherapy of the University Medical Center Mainz, in the period of June-August 2021, were asked to fill out the BPS and MSBS at the beginning of their inpatient treatment (initial rating). The MSBS referred to the patients’ current feelings while filling out the questionnaire. Participation was voluntary and did not affect the therapy of the participating patients. In the aforementioned period, we recruited a sample of *n* = 102 inpatients with different diagnoses who gave their consent to participate. If the patients were still under treatment 5–7 days after admission, they were asked to fill out the MSBS a second time (follow-up rating). This follow-up was only completed by approximately half of all inpatients who completed the initial rating. Further clinical data was collected from the patients, including age, gender, the main ICD-10 diagnosis leading to the current inpatient treatment, and duration of inpatient therapy (used as a proxy for therapy outcome). To quantify the therapy duration for all inpatients, the data analysis was started after the last inpatient of the study was released from the clinic. The demographic and clinical characteristics of all patients are summarized in Supplementary Table 2.

### Statistical analysis

All analyses were conducted using the MATLAB^®^ statistics and machine learning toolbox (The Mathworks Inc., Natick, Massachusetts, USA, version R2022a). All data was pseudonymized before analyzing.

#### Patient groups

To test for differences in boredom and therapy outcomes between different diagnosis groups, all participating inpatients were grouped according to the main ICD-10 diagnosis, which had led to their current hospitalization. We grouped inpatients into: psychotic disorders, addictions, depressive disorders, borderline personality disorder, and a category of all other psychiatric diagnoses. The different ICD-10 codes, which were assigned to each group, are reported in Supplementary Table 2. Note that drug-induced psychosis was assigned to the psychotic as well as addictive category.

#### Questionnaires

The self-reported data was analyzed by computing the sum score for each questionnaire. Subjects that accidentally skipped single items of a questionnaire or subscale were excluded from the respective analysis. This exclusion explains deviations from the total number of recruited participants/patients and the reported n of the respective analysis (e.g. see Fig. 3).

We aimed to investigate differences in state boredom between the different diagnosis groups within the inpatient cohort and to compare it to the healthy control cohort. Therefore, we first compared the summed state boredom ratings (MSBS) of each subgroup of inpatients with the healthy control cohort using a two-tailed Wilcoxon ranked sum test. In order to test for unspecific differences across the diagnosis groups, we conducted a Kruskal–Wallis test for the inpatient data only.

To compare the state of boredom of patients over the duration of their inpatient treatment, we conducted Wilcoxon signed-rank tests for all patients who completed the initial as well as the follow-up MSBS rating. This procedure was repeated separately for all diagnosis groups and for the pooled data of all patients, grouping them according to the condition of their inpatient treatment (open vs. closed ward).

#### Correlation analyses

**Fig. 1:**
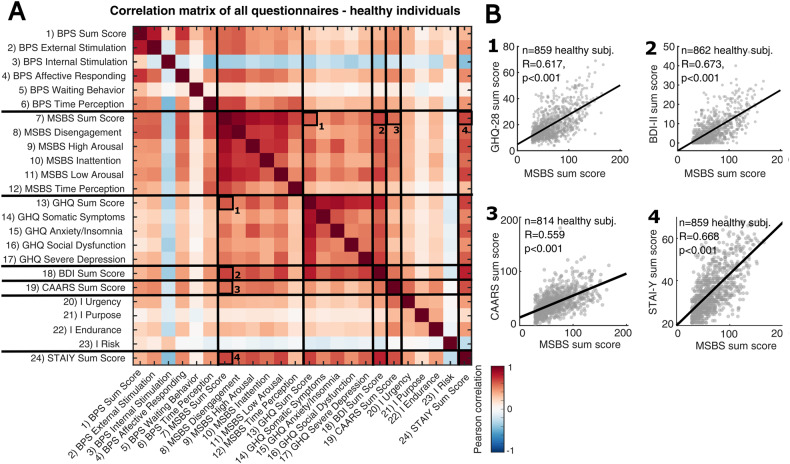
Boredom is related to psychopathological symptoms in a cohort of healthy individuals. **A** Pearson correlation matrix of various psychometric self-report assessments in a sample of *n* = 883 healthy individuals. The numbers indicate selected correlations of state boredom to psychopathological symptoms, which are further depicted in **B**. **B** Correlation scatter plots of state boredom and selected questionnaires reflecting psychopathology. State boredom is robustly correlated to 1: general symptoms of impaired mental health (General Health Questionnaire), 2: depressive symptoms (Beck’s depression inventory), 3: attentional deficits and hyperactivity (Conner’s Adult ADHD Rating Scale), 4: anxiety (State-Trait Anxiety Inventory). The black line indicates the linear fit. Lower *n* values for the correlation analyses result from the exclusion of subjects who incompletely filled out the questionnaires.

For the healthy cohort of individuals, we conducted an exploratory correlation analysis, testing the Pearson correlation of pairwise combinations of the participants’ psychometric ratings. We reported the raw correlation coefficients (see Fig. 1A) and furthermore tested the significance of these correlations by applying a Bonferroni correction for multiple testing (the correlations significant after the Bonferroni correction are detailed in Supplementary Fig. 1).

For the inpatient cohort, we conducted Pearson correlation analyses in order to investigate the relation between self-reported state boredom (MSBS), trait boredom (BPS), and the course of inpatient therapy (measured as the duration of stay in the hospital).

#### Multiple linear regression analysis

To test the effect of psychometric boredom scores, diagnosis groups, and the effect of treatment on open vs. closed wards on therapy duration, we conducted a multiple linear regression analysis. Here, we regressed the therapy duration of patients with initial and follow-up MSBS scores and BPS scores as steady parameters, and binary logical parameters for treatment on closed wards and the diagnosis groups (psychotic disorders, addictions, borderline personality disorder or depression, taking a value of 1 in the case of a certain diagnosis/treatment on a closed ward or a 0 in the case of absence). Together, this model formulates as:

$$\begin{array}{l}{therapy}\,{duration}={MSB}{S}_{{initial}}\,{\beta }_{1}+{MSB}{S}_{{follow}-{up}}\,{\beta }_{2}\\\qquad\qquad\qquad\qquad+\,{BPS}\,{\beta }_{3}+\,{wasOnClosedWard}\,{\beta }_{4}+\,{isPsychotic}\,{\beta }_{5}\\\qquad\qquad\qquad\qquad+\,{isAddicted}\,{\beta }_{6}+\,{isBorderline}\,{\beta }_{7}+{isDepressive}\,{\beta }_{8}\end{array}$$

Here, we only used data of *n* = 46 inpatients who completed all psychometric assessments yielding a complete dataset for the regression (statistics of the regression are shown in Supplementary Table 3). We furthermore tested the contribution of each parameter $j$ to predicting therapy duration by computing the absolute value of the respective regression term:

$${contributio}{n}_{j}={{\rm{|}}x}_{j}\,{\beta }_{j}{\rm{|}}$$

The contribution was computed with the respective parameter scores (${x}_{j}$, e.g. ${MSB}{S}_{{initial}}$) of each patient and compared across parameters.

## Results

### State boredom is associated with symptoms of psychopathology in healthy humans

We first sought to establish a reference dataset of state and trait boredom under non-clinical conditions and to explore the association of state and trait boredom with mild symptoms of psychopathology. Accordingly, we recruited a sample of 883 healthy human participants who filled out self-report assessments of state boredom (Multidimensional State Boredom Scale, MSBS), trait boredom (Boredom Proneness Scale) as well as psychometric questionnaires regarding general mental health (General Health Questionnaire, GHQ-28), depressive symptoms (Beck’s Depression Inventory II, BDI-II), symptoms of adult attentional deficit hyperactivity disorder (Conner’s Adult ADHD Rating Scale, CAARS-S:L), impulsivity (impulsivity questionnaire, I-8) and anxiety (State Trait Anxiety Inventory, STAI-Y) (see Methods). The recruited sample had an average age of 41.5 years (±SD 11.7), comprised predominantly of females (66.4%), and reported no active neuropsychiatric disorders (for demographic details see Supplementary Table 1).

With the various boredom-related and health-related psychometric assessments, we then conducted an exploratory correlation analysis (Fig. 1A). In line with previous studies (e.g. summarized in ref. [6]), we observed positive correlations of boredom ratings to various indicators of psychopathology in the sample of healthy individuals. Interestingly, the association with psychopathology was particularly strong for state boredom rather than trait boredom, showing a robust correlation to general mental health problems, symptoms of depression and ADHD as well as anxiety (Fig. 1B). Most of the correlations of boredom with psychopathological symptoms were robust against correction for multiple testing (Supplementary Figure 1), thereby corroborating their reliability.

Taken together, this correlation analysis shows that not only is trait boredom related to psychopathological symptoms in healthy humans, but also state boredom. This strong correlation for state boredom measured by the MSBS makes it a psychometric feature that could also be implicated in clinical conditions.

### Increased levels of boredom in a broad cohort of psychiatric inpatients

**Fig. 2:**
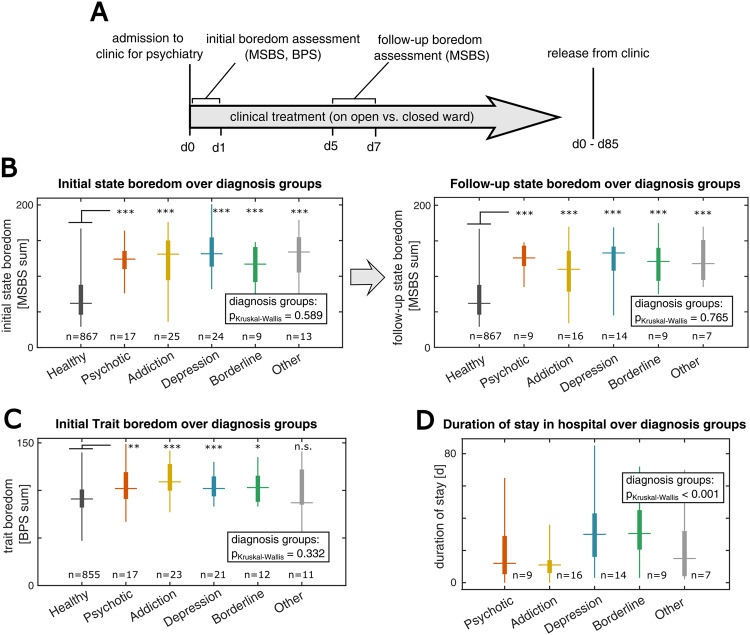
State boredom during hospital care in a broad cohort of psychiatric patients. **A** Schematic of the study flow. For a period of several weeks, all inpatients admitted to a psychiatric care clinic were screened for state and trait boredom at the beginning of their treatment (initial MSBS, BPS). Patients who stayed longer than 5d were surveyed again in respect to their state boredom (follow-up MSBS). Therapy duration ranged from zero (release on the day of admission) to 85 days. **B** Left panel: initial state boredom ratings for different patient groups (diagnosis groups) in comparison to the healthy sample (***p < 0.001 in a Wilcoxon rank sum test vs. healthy controls). Right panel: same analysis for the follow-up state boredom rating. **C** Equivalent analysis for the trait boredom ratings. Most diagnosis groups show higher trait boredom than healthy individuals (***p < 0.001, **p < 0.01, *p < 0.05 in a Wilcoxon rank sum test), but do not differ significantly between diagnosis groups. **D** Equivalent analysis for the duration of inpatient stay in the psychiatric hospital showing a significant difference between diagnosis groups.

In the next stage of our study, we sought to investigate the role of boredom in psychiatric patients and their inpatient therapy. We collected psychometric data from a broad cohort of inpatients (*n* = 102 patients) who were admitted to our psychiatric over a period of three months (Methods) and compared it to the reference data from the healthy cohort. At the beginning of the inpatient treatment, patients were asked to rate their initial state boredom (initial MSBS) and trait boredom (BPS). Then, after a period of 5–7 days, patients still under treatment were asked to rate their state boredom again (follow-up MSBS) (Fig. 2A). The patient cohort had an average age of 42 years (± SD 17.9), was made up of 45% females and hence was largely comparable to the aforementioned healthy sample (for demographic details see Supplementary Table 2). Therapy durations ranged between zero and 85 days.

To test for disorder-specific effects, we grouped patients according to the main diagnosis that led to their current admission. Specifically: Psychotic disorders (*n* = 21), addictions (*n* = 30), depression (*n* = 28), borderline personality disorder (*n* = 12), and other disorders (*n* = 14).

Comparing the state boredom ratings of the inpatient sample with the healthy controls, we found that all diagnosis groups showed increased levels of state boredom (Fig. 2B, Wilcoxon rank sum tests: *p* < 0.001 for all conditions). This elevation of state boredom held true for the initial as well as the follow-up assessment. However, we did not observe significant differences between the different diagnosis groups (Kruskal–Wallis tests: initial *p* = 0.589, follow-up *p* = 0.765). To corroborate these findings, we tested all subscales of the MSBS independently for differences between patients and healthy controls (Supplementary Figure 2). We observed that all subdimensions of state boredom were similarly raised in psychiatric inpatients, without relevant differences between diagnosis groups.

Carrying out the same analysis for the trait boredom ratings, we observed an increase in BPS scores in most diagnosis groups. This increase was less pronounced than that of state boredom, however (Fig. 2C, Wilcoxon ranked sum test: 4 of 5 conditions with *p* < 0.05). Between the various diagnosis groups, we did not find significant differences in trait boredom (Kruskal–Wallis test: *p* = 0.332).

To test the inpatient cohort for differences in treatment efficacy, we compared the inpatient treatment duration (measured as days spent in the psychiatric clinic) across all patient groups. Here, we found a significant group difference (Kruskal–Wallis test: *p* < 0.001), with the longest average stay for depressive patients and the shortest average stay for addictions. This indicates that diagnosis is a fundamental predictor of a patient’s treatment duration. For our next analyses, we operationalized inpatient treatment duration as a proxy for therapy complications.

### State boredom changes over inpatient treatment depending on the underlying mental disorder

**Fig. 3:**
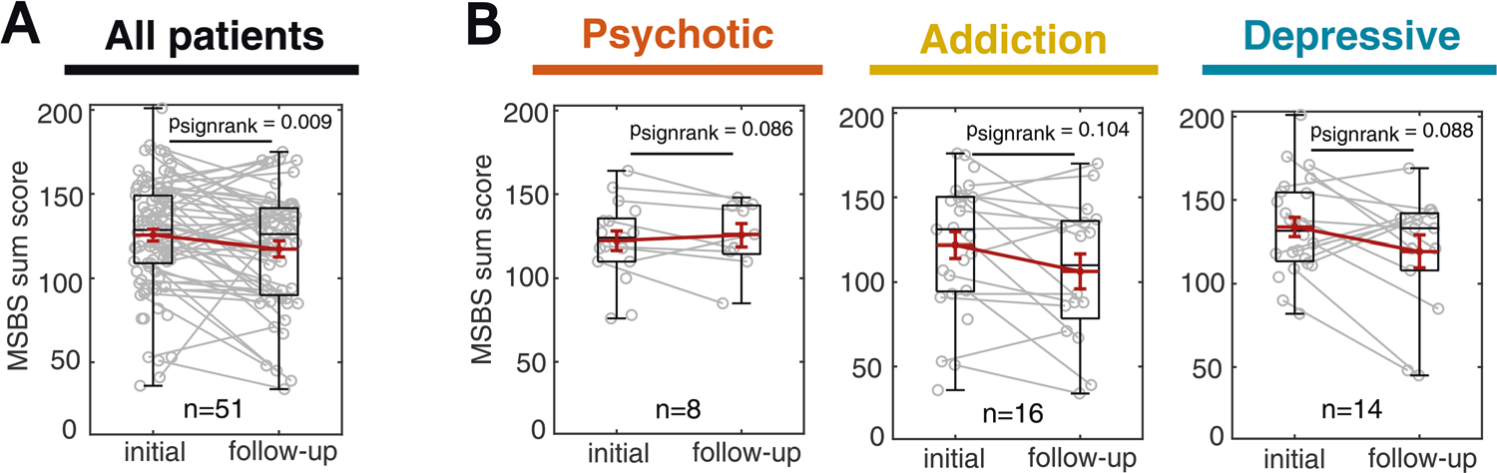
State boredom changes over inpatient treatment and depends on the underlying mental disorder. **A** State boredom (MSBS) at the initial and follow-up assessment (*n* = 51 of 102 patients that completed both assessments). **B** Equivalent plot as in **A**, grouping patients into the three largest diagnosis groups. Addicted and depressive inpatients show a trend of decreased boredom over the course of inpatient therapy, whereas psychotic inpatients tend to show increased state boredom over the course of inpatient therapy.

To characterize state boredom and its dynamics over the course of clinical treatment, we pooled the data from all patients and compared the initial state boredom ratings with the follow-up state boredom ratings (Fig. 3A). Here, we observed a significant reduction in state boredom over the first week of inpatient treatment (*n* = 51 patients with initial and follow-up MSBS rating, Wilcoxon signed-rank test: *p* = 0.009). Next, we conducted an analogous exploratory analysis for the three largest diagnosis groups: psychotic disorders, addictions, and depression (Fig. 3B). In addiction and depression, we found a tendency towards reduced state boredom over the course of therapy. However, psychotic patients tended to report higher state boredom after one week of therapy, indicating that state boredom is differentially modulated by different mental disorders and their treatment.

### Treatment on open versus closed wards is associated with differences in state boredom

**Fig. 4:**
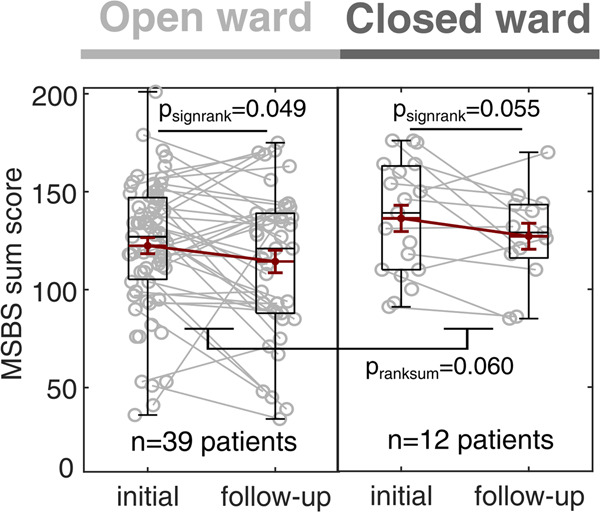
Treatment on open versus closed wards is associated with differences in state boredom. State boredom (MSBS) at the initial and follow-up assessment for patients grouped according to treatment in an open vs. closed psychiatric ward. In both groups, state boredom (MSBS) is reduced over the course of therapy, whereas the overall mean state boredom tends to be higher in patients treated on closed wards.

To investigate how the therapeutic environment of open versus closed psychiatric wards interacts with state boredom, we compared initial and follow-up MSBS ratings, grouping patients according to their ward of treatment (Fig. 4). All patients treated at least transiently on a closed ward during their inpatient stay (closed ward group) were compared to those patients who were solely treated on open wards (open ward group). For both groups, we found that state boredom declined over the course of one week (open ward: *n* = 39 patients, Wilcoxon signed-rank test: *p* = 0.049; closed ward: *n* = 12 patients, Wilcoxon signed-rank test: *p* = 0.055). Comparing the overall mean state boredom rating between both groups, we observed a trend of higher state boredom in patients treated in closed wards (Wilcoxon rank sum test: *p* = 0.060). Thus, state boredom affects inpatients on open and closed wards, where the environment of treatment potentially interacts with the boredom experience.

### State boredom after therapy initiation predicts prolonged clinical treatment

**Fig. 5:**
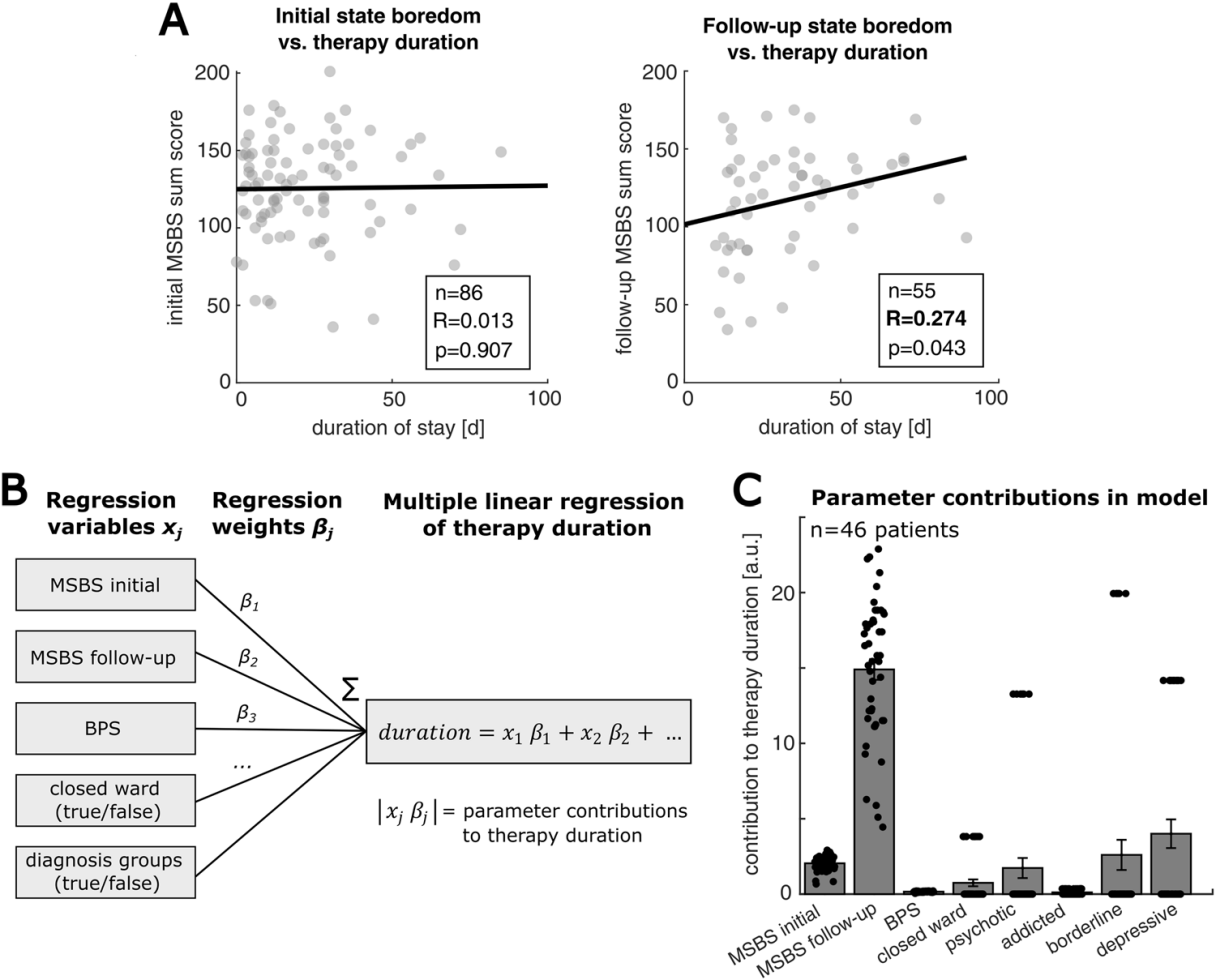
State boredom after therapy initiation predicts prolonged psychiatric therapy duration. **A** Left panel: Pearson correlation of initial state boredom and therapy duration. Right panel: Pearson correlation of follow-up state boredom and therapy duration, showing a positive association. **B** Schematic of a multiple linear regression model that we use to predict therapy duration with the following parameters: initial and follow-up MSBS scores, BPS score, treatment on closed wards, and the respective diagnosis group (psychotic, addiction, borderline personality disorder, depression). For the regression, we only use data of inpatients that yield a full dataset (*n* = 46 patients with complete dataset, for regression coefficient estimates see Supplementary Table 3). **C** Comparison of parameter contributions, expressed as the absolute value of the product of the respective parameter weight (*β*_j_) and the individual parameter score (*x*_*j*_). Residual state boredom after therapy initiation (follow-up MSBS) shows the highest mean contribution to therapy duration. Single dots show the parameter contribution for single patients. For the binary model parameters, such as diagnosis groups, only two values – a zero or the coefficient value – were possible, explaining the clustered datapoints. Vertical bars indicate the SEM).

Since we observed significant differences in therapy durations across our inpatient cohort and different associations of state boredom with diagnosis groups, we aimed to test if state boredom is a direct predictor of treatment outcome. Therefore, we pooled the data from all patients and correlated the initial and follow-up MSBS ratings with the duration of inpatient treatment (Fig. 5A). We did not find a significant correlation between initial state boredom and duration of therapy. However, we observed a positive correlation between follow-up state boredom and duration of therapy (*n* = 55 patients, *R* = 0.274, *p* = 0.043). This indicates that patients with high-state boredom after the initiation of inpatient therapy have a greater risk of undergoing complicated and prolonged treatment.

Although trait boredom ratings were positively related to initial state boredom (Supplementary Figure 3A), we did not observe a significant correlation of trait boredom with therapy outcome measured as the duration of inpatient stay (Supplementary Figure 3C).

We next sought to quantitatively compare the predictive power of state boredom on therapy duration with other predictive factors such as diagnosis groups. Accordingly, we conducted a multiple linear regression (Methods) to model individual therapy duration with state and trait boredom scores (initial and follow-up MSBS, BPS), the form of treatment (open vs. closed ward), and the diagnosis group (psychotic disorder, addiction, borderline personality disorder, depression) (Fig. 5B, regression statistics in Supplementary Table 3). We used the estimated parameter weights to compute the contribution of each regression parameter to therapy duration (Methods). Comparing the contributions across parameters, we found that follow-up state boredom has the strongest effect on therapy duration, followed by the psychiatric diagnosis groups. Thus, state boredom during clinical therapy has important implications for the outcome of inpatients.

## Discussion

In this study, we apply psychometric assessments of boredom in a broad cohort of psychiatric inpatients as well as a healthy control cohort. We observe that boredom shows a robust association with diverse psychopathologies. We further find that differences in state boredom are related to different classes of mental disorders and different types of clinical environments. Lastly, we demonstrate that state boredom occurring during the course of inpatient therapy is associated with prolonged inpatient treatment.

Despite a high positive correlation between state and trait boredom (Fig. 1, Supplementary Figure 3, ref. [31]) indicating that highly boredom-prone individuals also tend to experience boredom at a higher intensity, state boredom particularly (rather than trait boredom) is linked to psychopathology in both healthy humans and psychiatric inpatients. This was surprising, given most previous research had focused on links between trait boredom and symptoms of mental burden [11, 12, 16, 22, 25, 26], thereby raising the question about the interaction of state boredom and trait boredom. State boredom is thought to be triggered by external causes like monotonous sensory stimulation [2, 9, 39] or internal causes like attentional deficits [5, 8, 40], leading to coping mechanisms to alleviate boredom, such as mind-wandering [41] or a search for external stimulation [1, 2]. Trait boredom, conversely, has recently been defined in terms of the general frequency of experiencing state boredom, determined by an individual’s degree of agency and self-efficacy [7]. Moreover, it has been reported that trait boredom is associated with ineffective ways of coping with state boredom [42]. In addition, psychopathologies can prompt further independent cognitive and emotional stressors, such as apathy or anhedonia [43], for example, thereby impairing an individual’s ability to cope with boredom effectively [8, 30]. Thus, trait boredom and psychopathology are related in the sense that they both affect the frequency of boredom occurrence, allowing for an accumulation of state boredom over time. Accordingly, the intensity of state boredom at a given moment is mainly determined by the cumulated shortcomings in boredom coping, and therefore strongly represents an individual’s current psychopathological burden.

The link we observe between boredom and psychopathology relies on correlational analyses and hence does not permit the inference of causal relations. However, the fact that symptoms of mild psychopathology are linked to increased boredom, even in healthy individuals, suggests that boredom can indicate a higher potential risk of developing psychopathology [8, 26, 27]. This idea is supported by the decline of state boredom over the course of inpatient therapy, suggesting that on average clinical improvement is associated with reduced boredom. Together with various studies that identified boredom as a notable mental stressor during quarantine and pandemic restrictions of the past years [44–46], these findings highlight boredom as a potential early indicator of mental disorders and severe psychopathology.

Applying a multiple linear regression model to predict individual therapy duration based on boredom scores, therapy environment, and diagnosis groups, we observed that particularly state boredom affects the outcome of patients, even to a similar extent as the patient’s diagnosis. This observation, although limited to the set of inpatients who were still under treatment one week after admission, demonstrates that state boredom constitutes a clinically relevant, fundamental factor in mental disorders. This is in line with an earlier study that reported a high prevalence of boredom on acute psychiatric wards, but did not find a relevant correlation between trait boredom and therapy duration [47]. Together, these findings spotlight state boredom as a major effector in clinical psychiatry.

We observed a trend of higher state boredom in closed psychiatric wards compared to open wards. This group difference could be due to stronger a priori psychopathology in the patients who were treated in closed wards. An alternative interpretation, however, is that boredom was systematically increased by the restrictive environmental conditions of closed wards, pointing to potential factors that could be addressed in order to mitigate boredom. A valid evaluation of the environmental conditions during inpatient treatment that modulate boredom experience would however require close-meshed assessments of boredom [8, 26, 27, 30]. Here, it should be noted that different self-report scales to assess boredom have been shown to reflect different aspects of psychopathology [48, 49], highlighting their methodological constraints. In order to overcome some of these limitations and complement established self-report assessments, recent work provides a behavior-based measure of boredom [2] that could facilitate the investigation of boredom in clinical settings and its outward implications.

Interestingly, we observed that state boredom is increased widely across different groups of psychiatric patients. Furthermore, patient groups differ in their trend of state boredom after one week of therapy: psychotic patients tended to report increased boredom, whereas addicted and depressive patients reported declined boredom. How can these aspects of broad increase and differential trends of boredom among different diagnosis groups be integrated in one framework? State boredom has recently been described as a signal of the need for information [2, 4, 50]. Here, information is defined globally, involving external stimuli that are processed by sensory systems, as well as internal stimuli like thoughts that are conveyed through the nervous system. In conditions of low information, boredom acts as a signal for an organism to increase the flow of information to the brain, either by behaviorally seeking external stimulation [1, 2, 9] (*external coping*), or by engaging in internal stimulation such as mind-wandering [41, 51] (*internal coping*). Both coping mechanisms, internal and external, can be impaired independently in different mental disorders, leading to increased state boredom. For instance, formal thought disorder, as a major psychopathological feature in depression or schizophrenia, has been shown to correlate to altered network connectivity [52, 53], which likely disturbs internal information flow, hence impairing internal boredom coping. Conversely, problems in sensory processing that can be observed in various mental disorders [54–58] could explain deficits in the reception of external information, thus affecting external boredom coping.

This framework enables the understanding of increased state boredom in psychiatric patients as a consequence of individual psychopathological patterns that differentially affect the ability to cope with a lack of information. Hence, the increase in boredom over therapy duration, which we observe in psychotic patients, could be explained by a reduction of productive psychotic symptoms during the initial treatment with a concurrent lack of possibilities to achieve meaningful environmental stimulation. Conversely, the trend of reduced state boredom after one week of therapy in depressive patients could reflect an improvement in internal boredom coping due to an enhanced flow of thoughts. In this context, the degree of state boredom that persists or accumulates during an inpatient stay can be interpreted as an indicator of the aggregate psychopathological burden of an individual and how well these problems are addressed by the current therapy, thereby explaining the association between inpatient boredom and treatment duration.

Our characterization of boredom as an affective signal of low internal or external information flow integrates into existing theories that characterize boredom as a consequence of lacking agency [7], lacking attention [5], or lacking sense of meaning [59], since all three constructs implicitly depend on effective transmission of information in the brain.

Furthermore, the notion that increased state boredom can result from different coping deficits, which are themselves evoked by various psychopathological constellations, indicates the need for nuanced and specific intervention strategies to reduce boredom in mental health care. Earlier work has explored some approaches to address boredom in clinical settings, including the engagement of patients in meaningful activities [27, 28, 30], environmental enrichment [60], as well as mindfulness training [61], thereby effectively reducing patients’ state of boredom and aggression [28, 61]. In addition, a direct therapeutical addressing of boredom via psychotherapy and pharmacotherapy has been suggested [14, 30, 62]. Ideally, any therapeutic intervention to reduce boredom will comprise multiple approaches in order to target specifically the causes of boredom in a patient on an internal and environmental level [27, 30, 47]. Our results suggest that a broad range of psychiatric patients would benefit from such interventions.

Taken together, we demonstrate that increased state boredom is involved in mild psychopathological symptoms in healthy humans as well as in severe psychopathology in psychiatric patients. We find that boredom is generally reduced over the course of inpatient therapy and that state boredom during therapy is predictive of therapy outcome. Thus, we identify state boredom as an important factor, intensified by various psychopathologies, that can affect and potentially complicate the treatment of mental disorders. Targeting state boredom therapeutically offers novel starting points to mitigate the burden of psychopathology in mental disorders and to improve clinical outcomes.

### Supplementary information

Additional information

## Data Availability

The data of this study and the code to analyze it are available from the corresponding authors upon reasonable request.
