# Supplementary material for: High state boredom vastly affects psychiatric inpatients and predicts their treatment duration
Source: Transl Psychiatry. 2023 Nov 16;13:350. doi: 10.1038/s41398-023-02650-9 (PMC10654381; doi:10.1038/s41398-023-02650-9)
Supplement: Supplementary file 1 — Additional information [file 41398_2023_2650_MOESM1_ESM.pdf]

High state boredom vastly affects psychiatric inpatients and predicts their treatment duration

Johannes P.-H. Seiler[1,*], Katharina Zerr[2,3,*], Simon Rumpel[1,**], Oliver Tüscher[2,3,4,**]

[1] Institute of Physiology, University Medical Center of the Johannes Gutenberg University Mainz, Hanns-Dieter-Hüsch-Weg 19, 55131 Mainz, Germany

[2] Department of Psychiatry and Psychotherapy, University Medical Center of the Johannes Gutenberg University Mainz, Untere Zahlbacher Straße 8, 55131 Mainz, Germany

[3] Leibniz Institute for Resilience Research, Wallstraße 7, 55122 Mainz, Germany

[4] Institute of Molecular Biology, Ackermannweg 4, 55128 Mainz, Germany

[*] First authors that contributed equally

[**] Last authors that contributed equally

Correspondence concerning this article should be addressed to Johannes Seiler, Institute for Physiology, University Medical Center of the Johannes Gutenberg University Mainz, Hanns-Dieter-Hüsch-Weg 19, 55131 Mainz, Germany. E-mail: johseile@uni-mainz.de

Additional information

Supplementary Figure 1

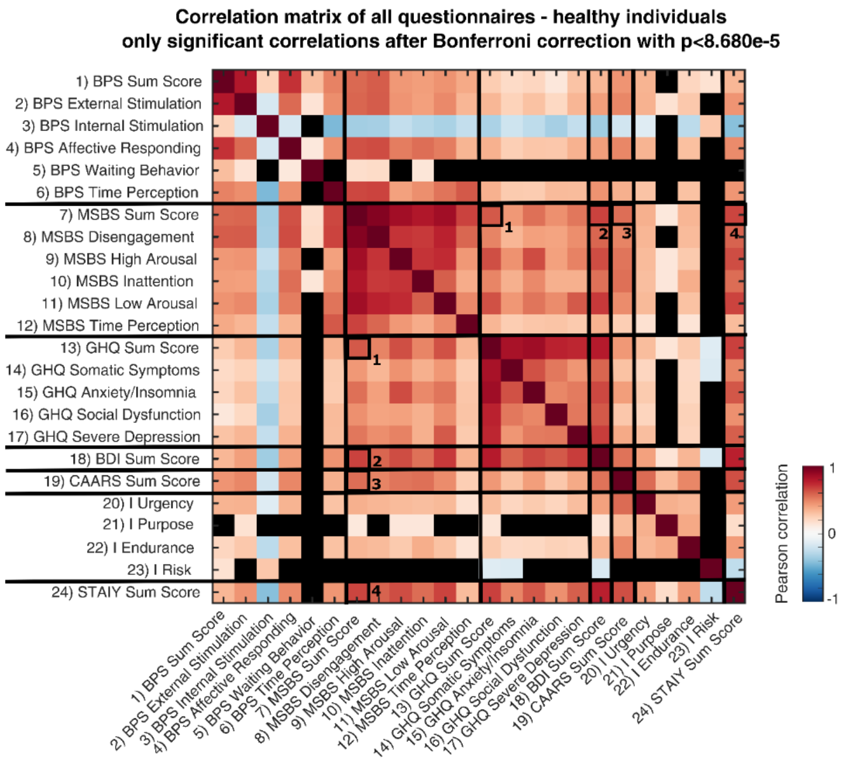

Supplementary Figure 1 – Correlation matrix of psychometric assessments controlled for multiple testing: Pearson correlation matrix of various psychometric self-report assessments in a sample of n=883 healthy individuals as also shown in Figure 1A. Here, each element of the matrix is controlled for statistical significance by applying a Bonferroni-corrected p threshold of p=8.680exp(-5) in order to account for multiple testing. Unsignificant elements are overshadowed by black rectangles.

Supplementary Figure 2

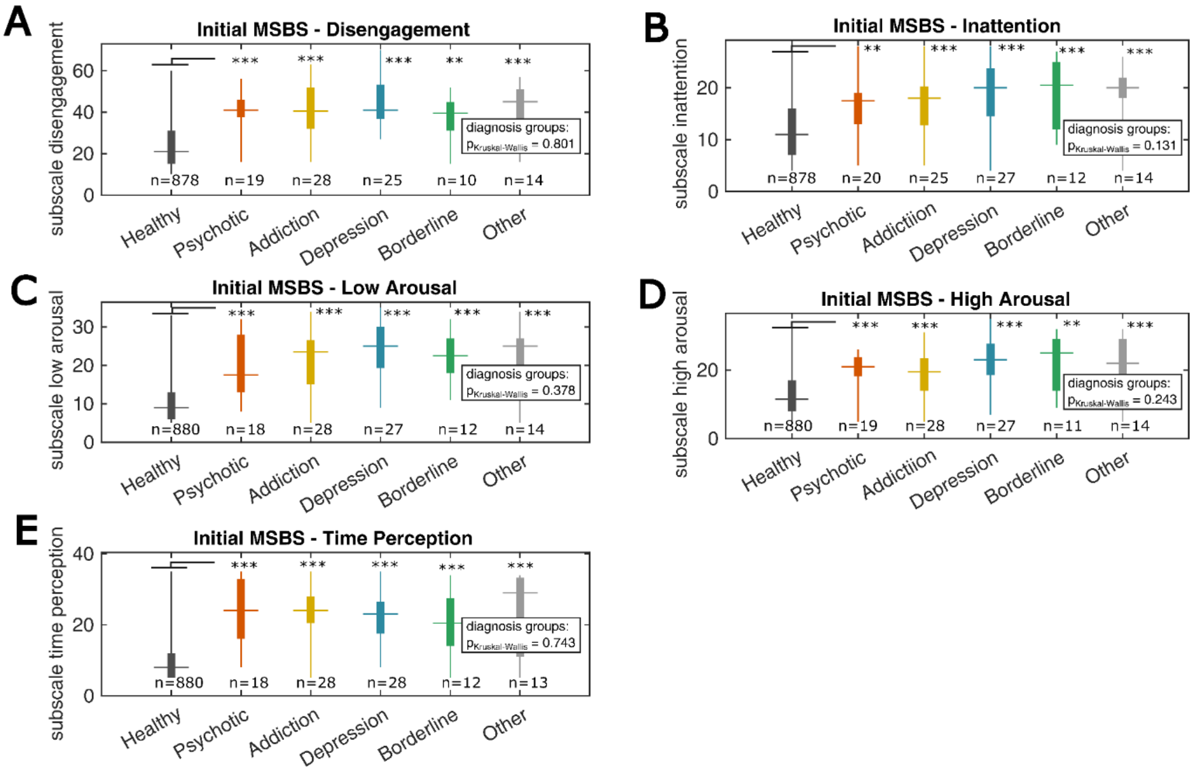

Supplementary Figure 2 – Subdimensions of state boredom ratings between healthy and inpatient cohort: (**A**) Disengagement subscale rating of the initial state boredom assessment for different inpatient groups in comparison to the healthy sample from Figure 1 (***: p<0.001, **: p<0.01, *: p<0.05 in a Wilcoxon rank sum test vs. healthy controls). (**B-E**) Equivalent analyses for the subscales Inattention, Low Arousal, High Arousal and Time Perception. All subdimensions of state boredom are increased relative to the healthy control group.

Supplementary Figure 3

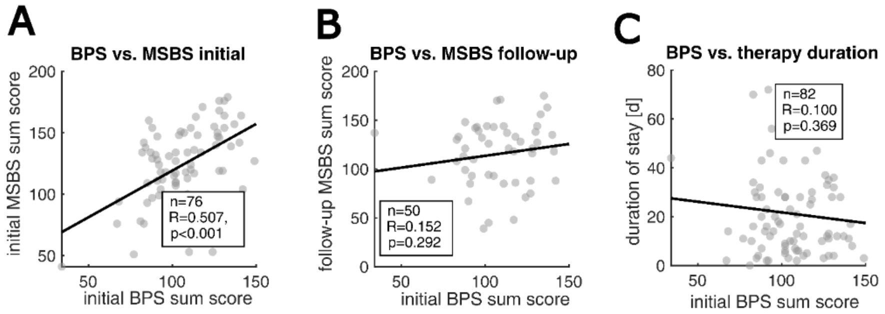

Supplementary Figure 3 – Correlation of trait boredom, state boredom and inpatient therapy duration: (**A**) Pearson correlation scatter plot of initial state boredom and trait boredom ratings for the inpatient cohort. In line with the healthy controls, this analysis reveals a positive association. (**B**) Equivalent analysis for the follow-up state boredom ratings vs. trait boredom ratings. (**C**) Equivalent analysis for the trait boredom ratings and inpatient therapy duration. Black lines indicate the linear fit.

Supplementary Table 1

	Healthy cohort (n = 883)
**Gender**	
Male	297 (33.6%)
Female	586 (66.4%)
**Age** (years)	
Mean	41.5
Standard deviation	11.72
**Home continent/country**	
Europe/Germany	808 (93.4%)
Europe/other	34 (4.0%)
Asia	17 (2.0%)
North America	2 (0.2%)
Middle America	1 (0.1%)
South America	2 (0.2%)
Africa	1 (0.1%)
**Psychiatric disorder in family**	
Yes	285 (32.3%)

Supplementary Table 1 – Demographic characteristics of the healthy control cohort

Supplementary Table 2

	All patients (n=102)	Psychotic (n=21)	Addiction (n=30)	Depressive (n=28)	Borderline (n=12)	Other (n=14)
**Included ICD-10 codes**	all	F06.2, F12.5, F19.5, F20.0	F10.0, F10.1, F10.2, F10.3, F10.4, F10.6, F11.2, F12.1, F12.2, F12.3, F12.5, F13.2, F15.1, F15.2, F15.7, F17.2, F19.1, F19.2, F19.5	F31.5, F32.2, F33.1, F33.2, F33.3	F60.31	F03, F06.3, F06.7, F31.2, F31.6, F40.1, F40.2, F41.0, F41.1, F42.0, F42.4, F43.0, F43.1, F43.2, F44.5, F45.41, F50.2, F60.4, F60.7, F71.1, F84.5, F90.0
**Gender**						
Male	56 (54.9%)	15 (71.4%)	24 (80%)	9 (32.1%)	3 (25%)	8 (57.1%)
Female	46 (45.1%)	6 (28.6%)	6 (20%)	19 (67.9%)	9 (75%)	6 (42.9%)
**Age** (years)						
Mean	42.2	42.0	44.0	48.0	29.1	37.6
SD	17.9	14.8	15.5	21.6	8.1	18.3
**Treatment on closed ward**						
Yes	23 (22.6%)	11 (52.4%)	8 (26.7%)	1 (3.6%)	0 (0%)	4 (28.6%)
**Therapy duration** (days)						
Mean	22.5	18.8	12.5	30.8	33.8	20.1
SD	18.7	18.9	8.5	19.3	21.7	19.5

Supplementary Table 2 – Demographic characteristics of the inpatient cohort

Supplementary Table 3

Parameter	Coeff. β	SE	t-stat.	p-val.
MSBS initial	0.016	0.099	0.164	0.870
MSBS follow-up	0.131	0.070	1.857	0.071
BPS	0.001	0.113	0.013	0.990
Was on closed ward	-3.818	6.464	-0.591	0.558
Is psychotic	13.270	9.122	1.455	0.154
Is addicted	0.355	7.544	0.047	0.963
Is Borderline	19.937	8.643	2.307	0.027
Is depressive	14.173	7.228	1.961	0.057
Root Mean Squared Error = 14.7				

Supplementary Table 3 –Multiple linear regression of therapy duration with psychometric and clinical parameters: n=46 patients with complete dataset. SE: squared error.
